# Maternal and Neonatal Urinary Iodine Status and its Effect on Neonatal TSH Levels in a Mildly Iodine-Deficient Area

**DOI:** 10.4274/Jcrpe.997

**Published:** 2013-05-30

**Authors:** Arzu Kutlu Yaman, Fatma Demirel, Bahri Ermiş, I. Etem Pişkin

**Affiliations:** 1 Beypazarı State Hospital, Department of Pediatrics, Ankara, Turkey; 2 Ankara Child Disease Hematology and Oncology Training Hospital, Department of Pediatric Endocrinology, Ankara, Turkey; 3 Sakarya University, Medical School, Department of Neonatology Unit, Sakarya, Turkey; 4 Bülent Ecevit University, Medical School, Department of Pediatrics, Zonguldak, Turkey

**Keywords:** Urinary iodine, maternal, neonatal, screening, hyperthyrotropinemia

## Abstract

**Objective:** Iodine deficiency and excess are the most important factors that affect screening and recall rates of congenital hypothyroidism. The purpose of this study was to investigate the urinary iodine status in newborns and their mothers and its effects on neonatal thyroid-stimulating hormone (TSH) levels in a mildly iodine-deficient area.

**Methods:** A total of 116 newborns and their mothers were included in the study. Urinary iodine levels were measured from healthy mothers and their babies on the 5th day following birth. Neonatal TSH levels were screened, and TSH and free thyroxine (fT4) levels were measured on the15th day in the recall cases. T4 treatment was started in infants with high TSH and low fT4 levels. These measurements were repeated on the 30th day in these newborns.

**Results:** Ninety-nine percent of the mothers included in the study were using iodized salt. The median urinary iodine level in the newborns was 279 µg/L, while it was 84 µg/L in their mothers. The rate of iodine deficiency among the mothers was 56.8%, and the rate of iodine excess was 8.6%. This rate was 10.3% for iodine deficiency and 61.2% for iodine excess in the newborns. The recall rate at the screening was 9.5% (n=11). The urinary iodine levels were above 200 µg/L in three newborns who had transient hyperthyrotropinemia.

**Conclusions**: Iodine deficiency was more frequently observed in nursing mothers, and iodine excess was more frequently seen in their newborns. The iodine excess noted in the newborns was attributed to the use of antiseptics containing iodine. The iodine excess leads to increases in recall rates, screening costs, and frequency of transient hyperthyrotropinemia.

**Conflict of interest:**None declared.

## INTRODUCTION

Sufficient iodine intake is necessary for the mother, fetus, and newborn to maintain normal thyroid functions. Iodine deficiency can cause infertility, endemic goiter, congenital anomalies, an increase in neonatal mortality rates, and mental retardation (1,2). Congenital hypothyroidism (CH) is one of the most important causes of preventable mental retardation, and was included in the neonate screening program in Turkey approximately 6 years ago (3). Iodine deficiency and excess are the primary factors that affect thyroid-stimulating hormone (TSH) levels and increase the recall rates for CH screening (4,5,6,7). In studies conducted following the national iodized salt program in Turkey, mild iodine deficiency was observed in the Western Black Sea region, particularly in the city of Zonguldak, and this area was accepted to be mildly endemic in terms of goiter prevalence and iodine deficiency (8,9). The purpose of the present study was to evaluate, 10 years after the initiation of the iodized salt program in Turkey, the iodine status of newborns and their mothers who lived in a city where mild iodine deficiency had been observed and to investigate its effects on neonatal TSH levels and thyroid functions. 

## METHODS

A total of 116 healthy term neonates and their healthy mothers, who were residents of Zonguldak, were included in the study. Mothers who had thyroid disorders, chronic illnesses, or took medication that might affect the thyroid function were excluded. The skin and genital regions of the mothers were cleaned with an antiseptic that contained povidone iodine before delivery, and povidone iodine was also used in normal spontaneous deliveries for cleaning the skin following episiotomy. An alcohol-based antiseptic that did not contain iodine was used for the umbilical regions of the babies.

Urinary iodine from spot urine samples was measured in the mothers and their babies on the 5^th^ day following birth. Blood samples were obtained from the babies using the heel-stick procedure for TSH screening between the 3^rd^ and 5^th^ days following birth, and again on the 15^th^ day for measuring TSH and free thyroxine (fT4) levels in the recall cases. In Turkey, the cut-off value for TSH screening is 15 mIU/mL, and babies with TSH values above the cut-off value are recalled on the 15^th^ day to check their TSH and fT4 levels (3); treatment is started if TSH levels are above 10 mIU/mL and fT4 levels are below or in the lower limit of normal. TSH and fT4 levels are repeated on the 30^th^ day after birth in these newborns. This group, in addition to newborns with permanent hypothyroidism, includes infants with transient hypothyroidism. Newborns with TSH levels above 10 mIU/mL and fT4 levels within the normal range are diagnosed as cases of transient hyperthyrotropinemia and are treated with low-dose Na-L-T4.

Written informed consent from the mothers and approval from the Ethics Committee of the hospital were obtained for the study.

Thyroid function tests were performed in the hospital biochemistry laboratory with an Immulite 2000 immunoassay system (Siemens Diagnostics, Terrytown, NY, USA) and kits of the same manufacturer using the chemiluminescence technique. Results were presented as mIU/mL for TSH and ng/dL for fT4.

Midstream urine samples were collected from the mothers. Urine bags were used for collection in the infants. The samples were transferred into 3-mL deionized tubes. All samples were stored in a deep freezer at -800C until the day of analysis. The samples were defrosted at room temperature prior to the analyses. Urinary iodine levels were measured with a Shimadzu UV-1601 spectrophotometer using the Sandell-Kolthoff reaction which utilizes colorimetric ceric-arsenic acid solutions. The urinary iodine levels were expressed as µg/L. An iodine level below 100 µg/L was considered as iodine deficiency, 100-199 µg/L as an iodine level within the normal ranges, and 200 µg/L and above as iodine excess (1,2,10).

## STATISTICAL ANALYSIS

Statistical Package for the Social Sciences (SPSS; Version 13.0) was used for statistical analyses. Kolmogorov-Smirnov test was used to determine normal distribution. Descriptive statistics were presented as mean ± standard deviation (SD) for normally distributed data, and as counts and percentages for categorical data. The relationship between the categorical variables was examined using the Chi-square test. Student’s t-test was used for the comparison of two groups with normally distributed variables, and the Mann-Whitney U-test was used for data not normally distributed. For the comparison of three or more groups, one-way analysis of variance (ANOVA) was used for normally distributed variables; otherwise, Kruskal-Wallis variance analysis was used. Results were evaluated with a confidence interval of 95%, and p<0.05 was considered statistically significant.

## RESULTS

Median values for urinary iodine excretion and iodine status of the mothers and their babies are presented in Table 1.

Although 99% of the mothers included in the study stated that they were using iodized salt, 56.8% were found to be iodine-deficient. On the other hand, most of the babies (61.0%) had iodine excess. It was found that 79.3% of the deliveries were caesarean sections (C/S) in the study group, and that only 20.7% (n=24) were normal spontaneous deliveries. There were no statistically significant differences between the mothers’ and babies’ urinary iodine levels according to method of delivery (p=0.731 and p=0.695, respectively).

The majority of the babies included in the study were breastfed (81.9%; n=95), while 16.4% were fed with breast milk and formula (n=19), and 1.7% were formula-fed only (n=2). There was no difference in urinary iodine excretion between these 3 feeding groups (p=0.821).

The mean TSH and fT4 levels of the neonates at screening and at recall visit (15th day) are given in Table 2. There were no significant differences between the groups. The recall rate of screening for CH was 9.5% (n=11) among all babies included in the study (Table 3). Two of the babies who were recalled had severe iodine deficiency, two had normal iodine excretion, and the rest had iodine excess. Na-L-T4 treatment was started in the three babies with a diagnosis of hyperthyrotropinemia (case numbers 9, 10, and 11 in Table 3) whose urinary iodine levels were above 200 µg/L. In these three newborns, the presence of thyroid gland was shown by thyroid ultrasonography scanning.

## DISCUSSION

The median urinary iodine levels of the mothers included in the present study indicate that the city of Zonguldak had the characteristics of a region with mild iodine deficiency. This finding was in line with the results of previous studies in the literature (8,9). Iodine deficiency in the mothers, despite the fact that almost all of them had used iodized salt during pregnancy, showed that the use of iodized salt during pregnancy and nursing periods did not meet their iodine requirements. Even though the use of iodized salt has become mandatory in Turkey since the initiation of the national program in 1998, studies have shown that the rate of iodine deficiency continued to be significantly high in sensitive populations, such as pregnant and nursing mothers (11,12,13,14). The iodine requirement increases in pregnant and nursing mothers and cannot be satisfied by using standard iodized salts. Iodine supplements are necessary during these periods. The mothers in this study used supplemental tablets that contained multivitamins and minerals recommended by their obstetricians, however, these products did not contain iodine. The use of vitamin-mineral combinations that contain iodine, alongside iodized salt, may help satisfy the increased need for iodine in pregnant and nursing mothers.

In the current study, 67% of the babies had iodine excess, and 10% had iodine deficiency. The excess iodine was believed to have originated from the use of povidone iodine for obstetric purposes during preparation for delivery. The use of povidone iodine for maternal skin cleaning causes iodine overload in the mother and excessive iodine transfer to the baby through the placenta and breast milk (5,7,15,16,17,18). Iodine excess in babies can also be caused by using povidone iodine for umbilical care or as eye drops (6,19). In the present study, iodine compounds were not used for umbilical or ocular care in the delivery room. Therefore, it was thought that the excess iodine in the babies was associated with high-level iodine transfer from the mother to the baby through the placenta and breast milk. Excess iodine in babies can raise costs by increasing recall rates of neonatal screening for TSH and cause transient hypothyroidism, hyperthyrotropinemia, and sometimes, severe hypothyroidism through the Wolff-Chaikoff effect (20,21,22,23,24). The recall rate in the current study was 9.5%, which was higher than expected. Iodine excess was determined in 63% of the recalled cases, while the ratio of iodine deficiency was 18%. Treatment was started in 3 infants in this series with diagnosis of hyperthyrotropinemia.

The majority of the mothers were found to have iodine deficiency despite the use of povidone iodine, and only a few of them had iodine excess. The presence of severe iodine deficiency in the majority of the mothers during gestation might have prevented iodine levels from increasing, although abundant levels of iodine are absorbed through the skin. Another possibility was that most of the iodine might have been eliminated via the urine until the 5th postpartum day when urinary iodine levels were measured in the mothers. Due to their immature kidneys, iodine clearance is low in newborns, and it takes time for the excess iodine to be cleared (24). Therefore, the iodine overload in our newborn cases was thought to be associated with their immature renal iodine excretion.

Iodine deficiency in the mother during the gestational period causes severe iodine deficiency in the fetus (1,2). Iodine overload in mothers and their babies in regions where iodine deficiency is observed may lead to a more intense Wolff-Chaikoff effect in the thyroid gland and higher TSH levels in newborns. Thyroid dysfunction due to iodine overload in some babies may necessitate long-term treatment (4,5,23,24). In the present study group, the high recall rate and the presence of iodine excess in three cases that were started on treatment for transient hyperthyrotropinemia led to a suspicion of the Wolff-Chaikoff effect associated with iodine overload.

There are some limitations of our study design. Urinary iodine concentration was not measured in the gestational period from mothers. This situation made it difficult to interpret the low iodine levels of lactating mothers. The other limitation is that the use of iodized salt was based on verbal information obtained from the mothers. Verbal information may have a low reliability, and measurement of iodine in the table salt used by these mothers could have provided us with more reliable information on the cause of the high iodine deficiency rate noted among lactating mothers in our study population.

In conclusion, iodine deficiency in nursing mothers in Turkey remains an important health problem. The use of iodized salt during pregnancy and lactation period appears to be insufficient in satisfying the iodine requirement. Recommending multivitamin tablets with iodine to pregnant and lactating women may provide a solution. Iodized antiseptics applied to mothers for cleansing the skin prior to delivery lead to iodine excess in newborns. Excess iodine in babies is considered a factor that causes increases in recall rates, screening costs, and frequency of transient hyperthyrotropinemia. Using compounds without iodine for antisepsis in the peripartum period might be an important step towards a solution to this public health problem.

## Figures and Tables

**Table 1:**
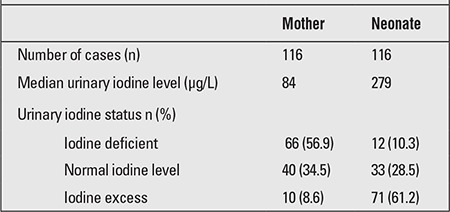
Urinary iodine status of the mothers and the neonates

**Table 2:**
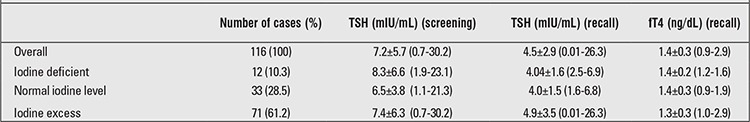
Mean thyroid-stimulating hormone (TSH) and free thyroxine (fT4) levels of the neonates in the study group

**Table 3:**
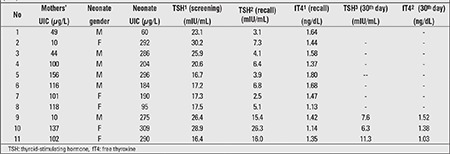
Urinary iodine status, thyroid-stimulating hormone (TSH) levels, and thyroid function tests of recalled neonates and urinary iodine concentrations(UIC) in their mothers
